# Aberrant expression of miRNAs in epilepsy

**DOI:** 10.1007/s11033-022-07188-5

**Published:** 2022-01-28

**Authors:** Soudeh Ghafouri-Fard, Bashdar Mahmud Hussen, Atefe Abak, Mohammad Taheri, Reza Jalili Khoshnoud

**Affiliations:** 1 Department of Medical Genetics, School of Medicine, Shahid Beheshti University of Medical Sciences, Tehran, Iran; 2 Department of Pharmacognosy, College of Pharmacy, Hawler Medical University, Erbil, Kurdistan Region Iraq; 3 Center of Research and Strategic Studies, Lebanese French University, Erbil, Kurdistan Region Iraq; 4 Men’s Health and Reproductive Health Research Center, Shahid Beheshti University of Medical Sciences, Tehran, Iran; 5 Institute of Human Genetics, Jena University Hospital, Jena, Germany; 6 Skull Base Research Center, Loghman Hakim Hospital, Shahid Beheshti University of Medical Sciences, Tehran, Iran; 7 Functional Neurosurgery Research Center, Shahid Beheshti University of Medical Sciences, Tehran, Iran

**Keywords:** miRNA, Epilepsy, Biomarker, Expression, Diagnosis

## Abstract

Epilepsy is manifested by intermittent convulsions and alterations in consciousness. This disorder has serious effects on daily functions and physical and mental health of affected patients. A variety of temporary irregularities in the function of brain can results in epilepsy. The molecular mechanism of epilepsy and the underlying causes of abnormal apoptotic responses in neurons, dysregulation of regenerative mechanisms in glial cells and abnormal immune reactions in the context of epilepsy are not clear. microRNAs (miRNAs) as important regulators of cell apoptosis as well as regenerative and immune responses have been shown to affect pathologic events in epilepsy. In the current review, we aimed at defining the role of miRNAs in the pathophysiology of epilepsy. We have listed dysregulated miRNAs in animal models of epilepsy and human subjects. miR-25-3p, miR-494, miR-139-5p, miR-101a-3p, miR-344a, miR-129, miR-298 and miR-187 are among down-regulated miRNAs in epilepsy. Moreover, expressions of miR-132, miR-146a, miR-181a and miR-155 have been reported to be increased in epilepsy. A number of genetic variants within miRNAs can affect risk of epilepsy. We discuss the role of miRNAs in the development of epilepsy.

## Introduction

Epilepsy is resulted from a variety of temporary irregularities in the function of brain due to an anomalous, extremely synchronous discharge of neuronal cells. Clinically, it is manifested by intermittent convulsions and alterations in consciousness, which has serious effects on daily functions and physical and mental health of affected patients [1]. Alterations in various gene patterns in the neurons can lead to the abnormal protein metabolism detected in the neurons of patients with this disorder [2, 3]. The molecular mechanism of epilepsy and the underlying causes of abnormal apoptotic responses in neurons, dysregulation of regenerative mechanisms in glial cells and abnormal immune reactions need to be clarified. microRNAs (miRNAs) as important regulators of cell apoptosis as well as regenerative and immune responses [4], are putative contributors in the pathogenesis of epilepsy. These small noncoding RNAs are approximately 19–22 nucleotides long and regulate gene expression through silencing mechanisms at posttranscriptional level [4]. It is estimated that more than one third of the human genome is under influence of regulatory roles of miRNAs [4]. Notably, nearly all regulatory mechanisms of expression of genes such as transcription factors as well as epigenetic factors have been found to act irregularly in the course of epilepsy [5]. As miRNAs can affect expression of transcription factors, dysregulation of miRNAs can influence epilepsy from different direct and indirect routes. In the current paper, we discuss the impact of dysregulation of miRNAs on development of epilepsy. We have searched PubMed and Google Scholar databases with key words "microRNA" or "miRNA" AND "epilepsy" or "seizure". After assessment of Abstracts and full texts of retrieved articles, we have included all relevant original papers in animal models and human subjects.

## Down-regulated miRNAs in epilepsy

### Refractory epilepsy

miR-139-5p is another miRNA with possible protective role against epilepsy. Expression of this miRNA has been found to be reduced in the sera of children with refractory epilepsy, parallel with up-regulation of expression of multidrug resistance-associated protein 1 (MRP1). The same expression pattern has been detected in the brain samples of rat models of refractory epilepsy. Functional studies have confirmed that MRP1 is targeted by miR-139-5p. Transfection of plasmids into the hippocampus of drug-resistant rats has verified the effects of miR-139-5p up-regulation or MRP1 silencing in reduction of neuron apoptosis, enhancement of neuron survival, and amendment of neuron injury. Thus, miR-139-5p/MRP1 axis can reduce resistance of refractory epilepsy to antiepileptic medications [6]. Expression of miR-34c-5p has also been reported to be decreased in patients with refractory epilepsy compared to controls. This miRNA targets the inflammation-related mediator gene HMGB1. Experiments in rat models of kainic acid (KA)-induced epilepsy have shown down-regulation of miR-34c-5p and up-regulation of HMGB1 and IL-1β in drug-resistant epileptic animals compared to drug-sensitive epileptic animals. Moreover, hippocampal neuron loss has been more prominent in drug-resistant epileptic animals. Thus, down-regulation of miR-34c-5p in refractory epilepsy aggravates neuroinflammatory responses, which exacerbates hippocampal neuron loss. These findings indicate that miR-34c-5p might be a possible noninvasive marker for refractory epilepsy [7]. miR-153 is another miRNA whose dysregulation is implicated in the pathogenesis of refractory epilepsy. This miRNA possibly acts through regulation of HIF-1α expression (8).

### Other types of epilepsy

Li et al. have examined expression levels of miR-15a-5p in serum samples of children with temporal lobe epilepsy (TLE). They have also cultured primary hippocampal cells in magnesium-deficient condition to simulate TLE. Their experiments have demonstrated down-regulation of miR-15a-5p in sera of children with TLE. Notably, miR-15a-5p has been confirmed to be an appropriate marker with proper specificity and specificity values for diagnosis of TLE in children. Moreover, magnesium-deficient condition has reduced expression levels of miR-15a-5p in hippocampal cells. On the other hand, up-regulation of miR-15a-5p has ameliorated TLE-associated decrease in cell viability, and attenuated the TLE-induced apoptosis. Therefore, miR-15a-5p has been suggested a promising marker for the detection of TLE in children [9].

**Fig. 1:**
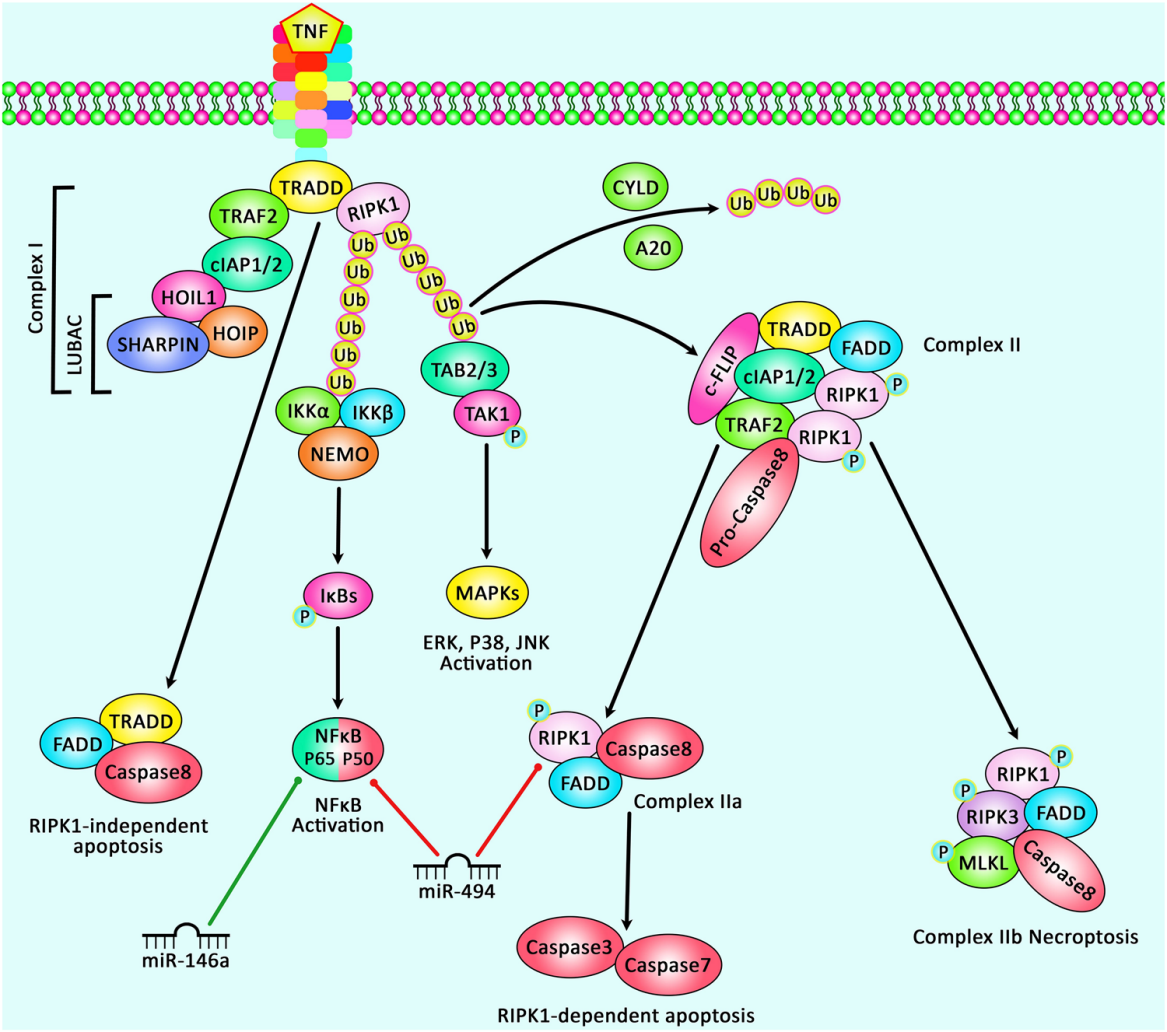
A schematic representation of the role of various miRNAs in modulating the NF-κB signaling cascade in epilepsy. After the binding of TNF to its receptor and stimulating it, TNFR1 could undergo a conformational alteration and recruit various proteins to create complex I, including TRADD, TRAF2/5, RIPK1, cIAP1/2, SHARPIN, HOIL-1, and HOIP. In complex I, the K63-linked ubiquitination of RIPK1 via cIAP1/2, leading to the binding of TAB2/3 and NEMO, modulating the recruitment of the TAK1/TAB complex and the IKKα/IKKβ/NEMO complex that could result in inducing the NF-κB cascade and cell survival. Eventually, the ubiquitinated RIPK1 could trigger the ubiquitination and proteasomal degradation of IkB, leading to nuclear transition of the released NF-kB and its upregulation. Besides, RIPK1 could regulate the overexpression of MAPKs, ERK, P38 and JNK that are induced via TNF-α. Destabilization of complex I leads to the creation of complex IIa, that includes TRADD, FADD and caspase-8. Furthermore, complex IIa contains the adaptor FADD, caspase 8, and RIPK1, and regulates the upregulation of caspase 8, then caspase 3, and caspase 7, resulting in apoptosis. When caspase 8 is suppressed, RIPK1 could merge with RHIM in RIPK3 to create complex IIb, creating RIPK3 oligomerization and autophosphorylation. Then, RIP3 could play an effective role in recruiting and phosphorylating MLKL, resulting in the necrosome [21, 22]. Growing evidences confirm that aberrant expression of miRNAs could have a crucial role in epilepsy. As an illustration, recent study has detected that upregulation of miR-494 could downregulate the expression level of RIPK1, triggering a suppression of the NF-κB signaling cascade and promotion of cell proliferation, and inhibition of apoptosis of hippocampal neurons in epilepsy, therefore attenuating the neuron injury and epilepsy development [12]. Furthermore, another research has illustrated that NF-κB could be modulated via miR-146a which has an important role in the pathogenesis of epilepsy development [16]

Another experiment in a rat model of epilepsy has shown that miR-21-5p can bind to STAT3. Expressions of caspase-3 and Bax have been higher, while expression of Bcl-2 has been lower in animals that received miR-21-5p inhibitor. miR-21-5p inhibitor has also resulted in loss of hippocampal neurons and induction of apoptosis in these cells, while suppression of STAT3 expression has led to opposite effects. Moreover, IL-6 levels have been higher in those received miR-21-5p inhibitor. Therefore, miR-21-5p is able to suppress expression of STAT3, decrease IL-6 levels and reduce loss of hippocampal neurons, thus protecting hippocampal neurons from deteriorating effects of epilepsy [10]. Table 1 shows the list of down-regulated miRNAs in epilepsy. Figure 1 illustrates the role of several miRNAs in epilepsy through regulating the NF-κB signaling pathway.

**Table 1:** Down-regulated miRNAs in epilepsy

microRNA	Samples	Assessed cell line	Interaction	Signaling pathway	Function	Reference
*miR-15a-5p*	Serum samples from 63 children with temporal lobe epilepsy (TLE) and 67 control subjects	Primary hippocampal neurons obtained from newborn rats	–	–	Its upregulation led to reduced apoptosis and augmented cell viability of hippocampal neurons	[9]
*miR-139-5p*	Serum samples from 26 children with refractory epilepsy, 35 children with newly diagnosed epilepsy, 12 children with traumatic injury of brain and 8 children with cerebrovascular malformation, male Sprague Dawley (SD) rats	–	MRP1	–	Its overexpression elevated drug sensitivity, diminished apoptosis and promoted survival in neuronal cells through targeting MRP1	[6]
*miR-34c-5p*	Plasma samples from 16 children with drug resistance, 8 children that are sensitive to anti-epileptic drugs and 8 controls, Male Sprague–Dawley rats	HEK-293 T	HMGB1	–	Was implicated in neuroinflammation and drug resistance through downregulation of HMGB1	[7]
*miR-21-5p*^a^	Male Sprague Dawley rats	HEK293T	STAT3	–	Diminished apoptosis rate and neuronal loss by targeting STAT3	[10]
*miR-25-3p*	40 male Sprague Dawley rats	Primary hippocampal neurons originated form rats	OXSR1↑	–	Its overexpression attenuated apoptosis, oxidative stress and constrained spontaneous recurrent epileptiform discharges by targeting OXSR1	[11]
*miR-494*	Male Sprague–Dawley (SD) rats	Hippocampal neurons from rats, 293 T	RIPK1↑	NF-κB signaling pathway	Its upregulation alleviated neuronal damage in hippocampal region, reduced apoptosis and augmented proliferation of neuronal cell by targeting RIPK1 and inactivation of NF-κB signaling pathway	[12]
*miR-139-5p*	Female Sprague–Dawley rats	Hippocampal neuron obtained from rats	Notch-1	Notch signaling pathway	Its upregulation decreased apoptosis and oxidative stress in hippocampal neurons by targeting Notch-1 and modulating Notch signaling pathway	[13]
*miR-101a-3p*	Sprague–Dawley (SD) rats	Primary hippocampal neurons obtained from rats	c-FOS↑	–	Its overexpression promoted cell survival, restrained apoptosis and autophagy by targeting c-FOS	[14]
*miR-344a*	Male Sprague–Dawley (SD) rats	–	–	–	Its upregulation resulted in decreased neuron damage and seizures	[15]
*miR-129*	30 adult Sprague Dawley (SD) rats	Primary hippocampal neurons obtained from rats	c-Fos↑	MAPK signaling pathway	Impeded epilepsy occurrence by downregulating c-Fos expression by suppression of MAPK signaling pathway	[16]
*miR-298*	–	HBMEC, U87-MG, HEK293T	P-gp↑	–	Sensitized HBMEC and U87-MG cells to antiepileptic drugs by targeting P-gp	[17]
*miR-187*	Hippocampal from 5 TLE patients and 5 normal subjects, male Sprague– Dawley (SD) rats	Primary hippocampal neurons obtained from rats	–	–	Its expression had inverse correlation with IL-10 secretion. Also its silencing increased IL-10 production, so can implicated in neuroinflammation and TLE pathogenesis	[18]
*miR-22*	Male C57Bl/6 mice	N2a, Primary hippocampal neurons from E18 embryonic mice	P2X7R	–	Its inhibition aggravated seizures by upregulation of P2X7R expression	[19]
*miR-153*	Brain tissues from 32 refractory mesial TLE (mTLE) patients and 18 controls, plasma samples from 56 mTLE patients and 101 healthy controls	Primary astrocyte isolated from rats	HIF-1α↑	–	Its downregulation might be implicated in pathogenesis of epilepsy through regulation of HIF-1α expression	[8]
*miR-204*	Sprague–Dawley rats	Primary hippocampal neurons	–	ERK1/2-CREB signaling pathway	Its upregulation suppressed epileptiform discharges by regulating expression of TrkB and modulation of ERK1/2-CREB signaling pathway	[20]

^a^Its expression has not been compared between normal and epileptic rats

### Up-regulated miRNAs in epilepsy

**Fig. 2:**
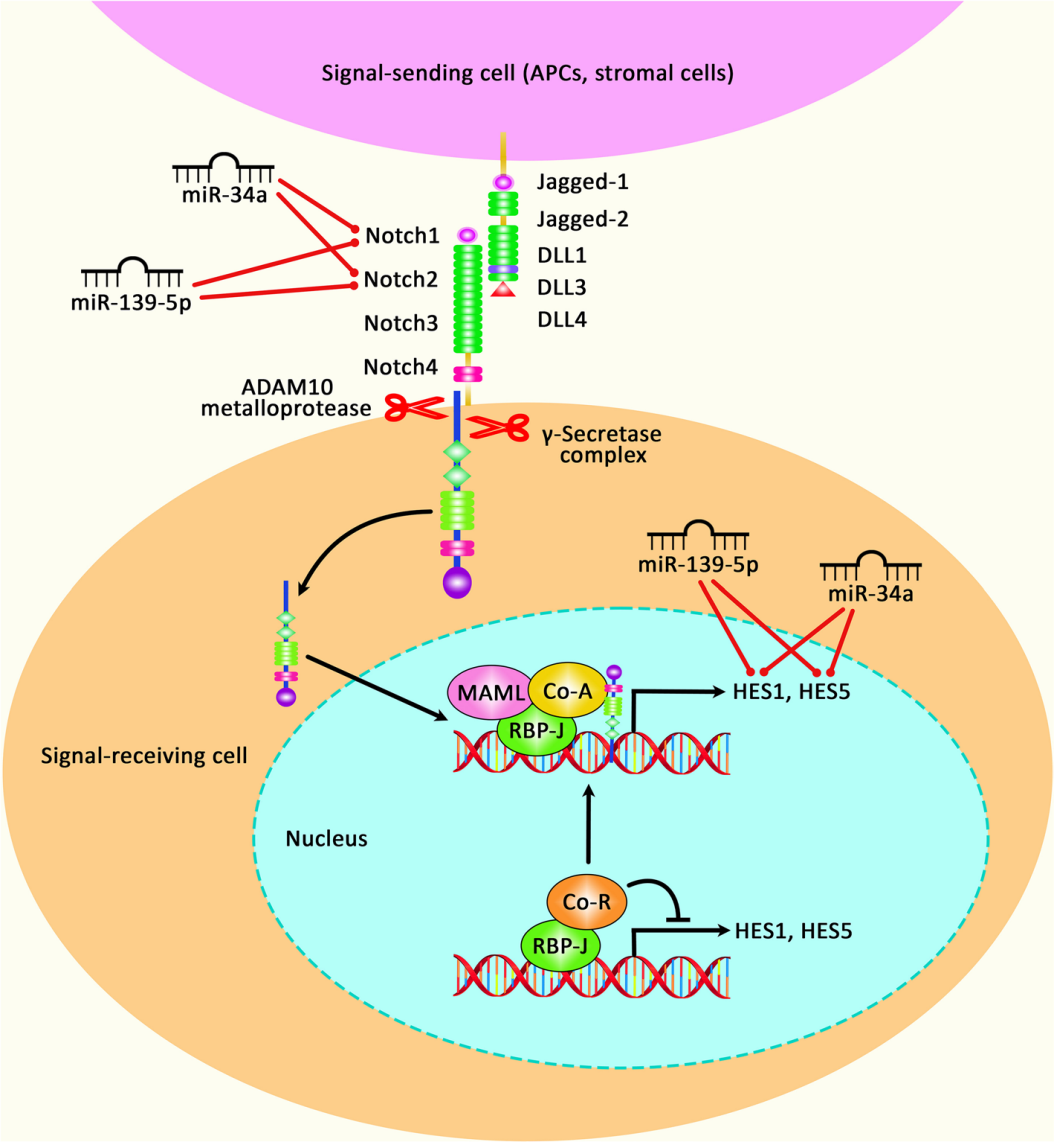
A schematic diagram of the role of several miRNAs in regulating the Notch signaling pathway in epilepsy. Interaction between Notch and Delta or Jagged that exist on the surface of signal-sending cells could modulate the cleavage of Notch protein via ADAM10 metalloproteases and γ-secretase to create NICD. NICD could transfer to the nucleus to create a heterotrimer with RBP-J and MAML to promote transcription of various target genes, including HES1 and HES5 [56]. Mounting evidence indicates that overexpression of various miRNAs could have a significant role in triggering epilepsy through Notch signaling cascade. Recent research has represented that downexpression of miRNA-34a could have a crucial role in suppressing epileptiform discharges via modulating Notch signaling and apoptosis in the rat hippocampal neuronal model of SREDs. In fact, SREDs could Induce the promotion of miR‑34a expression level and decrease of the expression of Notch signaling (including Notch1, Notch2, Hes1 and Hes5) [45]. In addition, another study has demonstrated that overexpression of miR-139-5p could suppress spontaneous recurrent epileptiform discharge-induced oxidative stress and apoptosis through modulating the Notch pathway [13]

Another experiment in epileptic rats has shown elevation of miR-103a and GFAP levels, higher quantity of apoptotic neurons, down-regulation of BDNF and reduction in the numbers of surviving neurons in hippocampal tissues of epileptic rats. Suppression of miR-103a has led to down-regulation of GFAP, up-regulation of BDNF and reduction in the number of apoptotic neurons, while enhancing the proportion of surviving neurons. Therefore, suppression of miR-103a results in the activation of astrocytes in hippocampus and amends neuronal damage in epileptic rats through regulation of expression of BDNF [23]. In addition, miR-27a-3p has been shown to be over-expressed in the hippocampal cells of epileptic rats and in KA-treated neurons. Notably, miR-27a-3p silencing has relieved epileptic seizures in animal models. In addition, miR-27a-3p silencing has suppressed apoptosis of hippocampal neurons in rat models of epilepsy, increased expression of Bcl2, and reduced levels of Bax and Caspase3. Moreover, miR-27a-3p silencing has efficiently decreased expressions of IL-1ß, IL-6, and TNF-α in hippocampal neurons. These effects are mediated through modulation of expression of MAP2K4, since this gene is a direct target of miR-27a-3p. miR-27a-3p silencing has also enhanced survival of KA-treated neurons, suppressed their apoptosis, increased expression of Bcl-2, and reduced expressions of Bax and Caspase3 through modulation of MAP2K4. Therefore, miR-27a-3p silencing protects against epilepsy-associated inflammatory responses and apoptosis of hippocampal neurons through influencing expression of MAP2K4 [24]. miR-132 is one of the most frequently upregulated miRNAs in animal models of TLE. This miRNA can affect functions of both neurons [25] and glial cells [26]. Expression of miR-132 has been found to be elevated in the hippocampal cells of human and rat epileptic subjects, principally in glial cells. Ectopic expression of miR-132 in human primary astrocytes has led to reduction of expression of a number of pro-epileptogenic genes, namely COX-2, IL-1β, TGF-β2, CCL2, and MMP3 [26]. The interaction between miR-132 and p250GAP/Cdc42 axis has been found as the uderlying mechanism of contribution of this miRNA in the epileptogenesis, based on the experiments performed in the hippocampal neurons cultures [25]. Another study has revealed a significant elevation in the levels of miR-132 and BDNF transcripts in the hippocampal neurons culture model of status epilepticus produced by Mg(2+)-deficient medium. Activation of TrkB.FL by pretreatment with BDNF has partially suppressed the Mg(2+)-free associated unremitting high-frequency epileptiform discharges, whereas up-regulation of miR-132 has aggravated epileptiform discharges. miR-132 has also been found to partake in the postepileptic augmentation of high voltage dependent calcium channels. Therefore, miR-132 has pro-epileptic effects via modulating BDNF/TrkB pathway in the hippocampal neuron culture model of status epilepticus [27]. miR-146a is another up-regulated miRNA in the course of epilepsy. Experiments in a rat model of TLE have shown up-regulation of miR-146a in the hippocampal tissues. miR-146a knock down has remarkably amended neuron injury and cell apoptosis in rat hippocampus, reduced MDA, IL-1β, IL-6, and IL-18 expressions and enhanced SOD levels in this tissue. Moreover, miR-146a silencing has reduced expressions of caspase-9, GFAP, Notch-1, and Hes-1 in the hippocampus of animal models of TLE. Functional studies have shown Notch-1 as the target of miR-146a. Thus, miR-146a silencing alleviates neuron injury in the hippocampus of animal models of TLE through inhibiting expression of Notch-1 [28]. Another study has demonstrated high levels of miR‑146a in the lithium‑pilocarpine- induced model of epilepsy. miR-146 silencing has led to reduction of IL‑1β, IL‑6 and TNF‑α levels. Moreover, expressions of P‑gp and p‑P65/P65 have been decreased after miR‑146a silencing, while expressions of Bcl‑2/Bax have been increased following this intervention [16]. miR-181a is another up-regulated miRNA in epilepsy. Its inhibition has resulted in protective effects against epilepsy, reduced apoptosis and decreased activation of microglia and astrocyte by upregulating SIRT1 [29]. Moreover, its silencing has constrained apoptosis in hippocampal neurons [30]. Expression levels of miR-21-5p and mTOR have been shown to be increased in rats during acute, latent, and chronic phases of epilepsy parallel with down-regulation of PTEN. In vivo suppression of miR-21-5p has led to down-regulation of mTOR and up-regulation of PTEN. miR-21-5p silencing has also reduced the quantity of abnormal spikes in EEG and diminished the neuron defects. Moreover, this intervention has ameliorated epilepsy-induced cognitive and memory damages in vivo. Targeting PTEN-mTOR axis by miR-21-5p has been identified as the molecular mechanism of participation of this miRNA in the pathogenesis of epilepsy [31]. Table 2 shows up-regulated miRNAs in epilepsy. Figure 2 represents the role of various miRNAs via regulating the Notch signaling cascade in epilepsy.

**Table 2:** Up-regulated miRNAs in epilepsy

microRNA	Samples	Assessed cell line	Gene/protein interaction	Signaling pathway	Association with clinical characteristics	Function	Reference
*miR-103a*	90 clean-grade and healthy Sprague–Dawley (SD) rats	Hippocampal neurons obtained from rats	BDNF	–	–	Its downregulation ameliorated neuronal injury and reduced apoptosis rate of neurons in epileptic rats	[23]
*miR-27a-3p*	Male Sprague–Dawley (SD) rats	Primary hippocampal neurons originated form rat	MAP2K4	–	–	Its inhibition resulted in decreased apoptosis rate of hippocampal neurons, elevated cell viability and alleviation of seizures through regulation of MAP2K4 expression	[24]
*miR-132*	Brain tissue specimens from 16 TLE patients and 10 controls, adult male Sprague Dawley rat	Primary fetal astrocyte from rats	–	TGF‐β pathway	–	Was implicated in pathogenesis of epilepsy through modulating TGF‐β pathway and regulation of pro‐epileptogenic factors	[26]
*miR-132*	Male C57BL/6 mice	Hippocampal neurons from mice	–	–	–	Its silencing inhibit seizures possibly through regulation of p250GAP/Cdc42 axis	[25]
*miR-132*	Sprague–Dawley rats	Primary hippocampal neurons from rats	–	BDNF/TrkB signaling	–	Aggravated epileptiform discharges and contributed to epileptogenesis by modulating BDNF/TrkB signaling	[27]
*miR-146a*	128 male Wistar rats	293 T cells	Notch-1	–	–	Its silencing ameliorated neuronal injury and reduced apoptosis rate through regulation of Notch-1 expression	[28]
*miR-146a*	Male Sprague–Dawley rats	–	–	NF‑κB pathway	–	Its silencing has protective effects against status epilepticus through modulation of NF‑κB pathway	[16]
*miR-146a*	Male Sprague–Dawley rats	THP-1, U373, SH-SY5Y	CFH↓	–	–	Its inhibition alleviated seizures by enhancing expression of CFH	[32]
*miR-181a-5p*	Male Sprague–Dawley rats	293 T cells, hippocampal neurons obtained from rats	SIRT1	–	–	Its inhibition has protective effects against epilepsy, reduced apoptosis and decreased activation of microglia and astrocyte by upregulating SIRT1	[29]
*miR-181a*	Brain tissues from 25 TLE children, male Sprague–Dawley rats	–	–	–	–	Its silencing constrain apoptosis in hippocampal neurons	[30]
*miR-155*	Brain tissue specimens from 12 TLE patients and 11 patients with malformation temporal vessels with no epilepsy history, plasma samples from 40 epileptic patients and 40 non-epileptic volunteers, male C57BL/6 mice	Primary microglia, astrocyte, neuronal cells obtained from C57BL/6 mice	–	–	–	Its silencing led to decrease in expression of pro-inflammatory cytokines and seizure frequency	[33]
*miR-155*	Brain tissues from 16 TLE patients and 10 control individuals, male Sprague–Dawley rats	Primary fetal astrocyte-enriched cell cultures obtained from human fetal brain tissue	–	–	–	Its inhibition led to reduction in expression of MMP3. So it can be implicated in pathogenesis of TLE and can be considered as potential therapeutic target	[34]
*miR-155*	Hippocampal tissue samples from 68 TLE patients and 42 temporal cortex tissues from control subjects, male Sprague–Dawley rats	HEK293, PC12	Sesn3 (is target of Rattus norvegicus miR-155 or rno-miR-155)	–	Hippocampal sclerosis	rno-miR-155 knockdown alleviated pathophysiological features of epilepsy and reduced apoptosis rate in rat hippocampus by regulating Sesn3 expression	[35]
*miR-155*	C57BL/6 mice	Primary neurons from rats	BDNF	–	–	Its silencing ameliorated seizures by modulating BDNF activation	[36]
*miR-135a-5p*	Brain tissues from 15 children with TLE and 15 control subjects	Primary hippocampal neurons from rats	CAAP1↓	–	–	Enhances apoptosis rate and reduces cell viability hippocampal neurons through inhibition of CAAP1	[37]
*miR-135a-5p*	–	BV2	SIRT1	–	–	Its inhibition suppressed apoptosis and enhanced proliferative ability in microglia by regulating expression of SIRT1	[38]
*miR‑128*	Male Sprague–Dawley rats	PC12	SIRT1	–	–	Induces apoptosis in PC12 cells through targeting SIRT1 and modulation of SIRT1/p53/Bax/Cytochrome c/caspase pathway	[39]
*miR-134*	Male Sprague–Dawley rats	–	–	–	–	Its silencing conferred reduced neuronal damage and constrained spontaneous seizures	[40]
*miR-134*	Male C57BL/6J mice	SH-SY5Y	Tulp1↓	–	–	Might be implicated in development of epilepsy and excitotoxicity in neurons by targeting Tulp1	[41]
*miR-134*	Male Sprague–Dawley (SD) rats	Primary neuron culture from rats, HEK293T	limk1	–	–	Its silencing could have neuroprotective effects by upregulation of limk1 and downregulation of cofilin	[42]
*miR-134*	Brain tissues from epilepsy patients and normal individuals, male C57BL/6 mice	SH-SY5Y	–	–	–	Its silencing had neuroprotective effects against seizures	[43]
*miR‑142*	96 male Wistar rats	293 T	PINK1	–	–	Its inhibition constrained apoptosis, attenuated apoptosis and promoted mitochondrial autophagy through modulating expression of PINK1	[44]
*miR-34a*	–	Primary hippocampal neurons obtained from Sprague–Dawley rats	–	Notch signaling pathway	–	Elevated apoptosis rate in neuronal cells and inhibited Notch signaling pathway	[45]
*miR-142-5p*	Male C57BL/6 mice,	293 T	Miro1↓	–	–	Its silencing lowered apoptosis and neuronal damage in hippocampal region and alleviated status epilepticus by targeting Miro1	[46]
*miR-141*	Wistar Han male rats	C6 glioma cell line	SIRT1	p53 signaling pathway	–	Potentiated apoptosis, constrained cell proliferation through targeting SIRT1 and modulation of p53 signaling pathway	[21]
*miR‐20a‐5p*	Male Sprague‐Dawley rats	293 T, PC12, primary hippocampal neurons	RGMa↓	–	–	Its silencing repressed axonal growth and branching of neuronal cells and impeded development of epilepsy by targeting RGMa and adjusting of RGMa/RhoA axis and synaptic plasticity	[47]
*miR-183*	85 male Sprague Dawley rats	Primary hippocampal neurons from rats	Foxp1↓	Jak/Stat signaling pathway	–	Its inhibition resulted in declined apoptosis rate, ameliorated hippocampal neuron injury and raised proliferation of neuronal cells by targeting Foxp1	[48]
*miR-200c-3p*	55 male Wistar rats	HEK-293 T	RECK↓	AKT signaling pathway	–	Its silencing repressed apoptosis in neuronal cells and lowered hippocampal neuron injury by targeting RECK and inhibition of AKT signaling pathway	[49]
*hsa-miR-1275 *(downregulated in CSF samples)	Serum samples from 11 patients with epilepsy of unknown etiology (EUE) and 10 healthy subjects, CSF samples from 6 EUE patients and 3 controls	U251	MECP2	–	–	Raised apoptosis rate and might be implicated in pathogenesis of EUE	[50]
*miR-23a*	Male C57BL/6J mice	293 T	ADAM10↓	–	–	Its silencing repressed spontaneous recurrent seizures by increasing expression of ADAM10	[51]
*miR‑155*	Blood samples from TLE patients and healthy volunteers	HT22	–	PI3K/Akt/mTOR signaling pathway	–	Contributed to apoptosis in hippocampal neurons and epilepsy development by modulating PI3K/Akt/mTOR signaling pathway	[52]
*miR-129-5p*	Naive mice	–	Atp2b4, Dcx	–	–	Its inhibition suppressed synaptic downscaling in neurons by modulating Atp2b4/Dcx axis and crosstalk with Rbfox	[53]
*miR-21-5p*	Male Wistar rats	–	–	PTEN-mTOR signaling pathway	–	Its silencing ameliorated memory impairment and cognitive problems and decreased neuronal loss by modulating PTEN-mTOR signaling pathway	[31]
*miR-203*	Hippocampal tissues from 6 epilepsy patients and 6 controls, male C57BL/6J mice	Neuro-2a, HeLa	GLRB	–	–	Its inhibition decreased frequency of seizures by regulation of GLRB expression	[54]
*miR-199a-5p*	Male Sprague–Dawley rats	–	–	–	–	Its silencing exerted neuroprotective effects by regulating SIRT1/p53 axis	[55]

### Diagnostic/prognostic role of miRNAs in epilepsy

Several miRNAs have been found to have potential applicability as diagnostic or prognostic markers in epilepsy. For instance, miR-15a-5p has diagnostic power of 0.908 with 82.5% sensitivity and 88.1% specificity in diagnosis of TLE children from healthy matched controls [9]. Expression of hsa-miR-134 has been found to be deceased in patients with mesial TLE (MTLE) but not in patients with focal cortical dysplasia (FCD) when compared to healthy subjects. hsa-miR-134 could separate MTLE patients from controls with diagnostic power of 0.75. This result has been validated an independent cohort of patients with MTLE including both refractory and drug-responsive patients. Therefore, hsa-miR-134 has been suggested as a marker for MTLE in an independent manner from their response antiepileptic drugs or existence of MRI signs of hippocampal sclerosis [57]. Another study has investigated the role of miR-27a-3p, miR-328-3p and miR-654-3p as putative circulating biomarkers for epilepsy diagnosis and prediction of outcome of surgical intervention in a cohort of MTLE with hippocampal sclerosis (MTLE-HS) including those with good surgical prognosis (Engel I) and those with unfavorable surgical prognosis (Engel III-IV). miR-27a-3p has not been validated as a circulatory marker for diagnostic or prognostic purposes. However, miR-328-3p could differentiate controls from Engel I, controls from Engel III-IV and controls from Engel I + Engel III-IV patients with diagnostic power values of 90.3%, 96.8% and 93.5%, respectively. In addition, miR-654-3p could differentiate controls from Engel I patients as well as patients with unfavorable from favorable surgical outcome with lower values [58].

Although the diagnostic power of several miRNAs has been assessed in epilepsy, a major drawback of the majority of studies is lack of confirmation of the obtained results in independent cohorts of patients. Moreover, only few miRNAs have been assessed in more than one study. For instance, miR-134 could differentiate patients with MTLE from controls with diagnostic power of 0.75 [57]. It could also predict development of drug-resistance with power of 0.61 [59]. Since studies in this regard are not ample, it is not possible to find the impact of sample size, variations in the methodology, or geographic origin of the cohorts studied on the obtained results.

Table 3 shows the diagnostic and prognostic role of miRNAs in epilepsy.

**Table 3:** Diagnostic/prognostic role of miRNAs in epilepsy

microRNA	Expression pattern	Samples	Diagnostic or prognostic biomarker	ROC curve analysis			Reference
				Sensitivity (%)	Specificity (%)	AUC	
*miR-145-5p*	Downregulated	Plasma samples from 40 refractory epilepsy patients and 42 normal subjects	Diagnostic biomarker (refractory epilepsy patients vs. healthy controls)	–	–	0.632	[60]
*miR-15a-5p*	Downregulated	Serum samples from 63 children with temporal lobe epilepsy (TLE) and 67 control subjects	Diagnostic biomarker (TLE patients vs. healthy controls)	82.5	88.1	0.908	[9]
*hsa-miR-134*	Downregulated	Plasma samples from 14 patients with mesial temporal lobe epilepsy (MTLE), 13 patients with focal cortical dysplasia (FCD) and 16 control individuals Another cohort of 65 MLE patients and 83 controls was considered as validation set	Diagnostic biomarker (MTLE vs. controls)	–	–	0.75	[57]
*miR-146*	Upregulated	Serum samples from 162 patients with focal impaired awareness seizures including 86 drug-resistant patients 76 drug-responsive patients	Prognostic biomarker (predicting development of drug-resistance)	–	–	0.640	[59]
*miR-134*	Upregulated		Prognostic biomarker (predicting development of drug-resistance)	–	–	0.617	
*miR-328-3p*	Upregulated	Serum samples from 28 patients with mesial temporal lobe epilepsy with hippocampal sclerosis (MTLE-HS) including 14 with good surgical prognosis (Engel I) and 14 with undesired surgical prognosis (Engel III–IV) and 11 control subjects	Diagnostic biomarker (Engel I patients vs controls)	78.60	100	90.3%	[58]
*miR-328-3p*	Upregulated		Diagnostic biomarker (Engel III-IV patients vs controls)	100	90.9	96.8%	
*miR-328-3p*	Upregulated		Diagnostic biomarker (epilepsy patients (Engel I + Engel III-IV) vs controls)	89.30	90.90	93.5%	
*miR-654-3p*	Upregulated (in Engel I patients compared to controls)		Diagnostic biomarker (Engel I patients vs controls)	57.10	100	74.7%	
*miR-654-3p*	Upregulated (in Engel I patients compared to controls)		Diagnostic biomarker (Engel I patients vs Engel III-IV patients)	57.10	84.60	73.6%	
*miR-146a* *miR-155* *miR-132*	Upregulated Upregulated –	Serum samples from patients with genetic generalized epilepsy (GGE) and 67 healthy volunteers	Diagnostic biomarker (GGE patients vs. healthy controls)	73	80	0.85	[61]
*miR-142*	Upregulated	Serum samples from 27 TLE patients including 10 drug-resistant and 17 drug-responsive and 20 healthy individuals	Diagnostic biomarker (drug-resistant patients as drug-responsive patients)	–	–	0.80	[62]
*miR-223*	Upregulated		Diagnostic biomarker (drug-resistant patients as drug-responsive patients)	–	–	0.75	
*miR-142* *miR-223*	Upregulated Upregulated		Diagnostic biomarker (drug-resistant patients as drug-responsive patients)	–	–	0.80	
*miR-451a*	Upregulated (in status epilepticus)	CSF samples from 29 TLE patients, 32 patients with status epilepticus (SE) and 40 control subjects	Diagnostic biomarker (TLE patients vs. SE patients)	–	–	0.91	[63]
*miR-21-5p*	–		Diagnostic biomarker (distinguishing SE patients from controls)	–	–	0.83	
*miR-3613-5p*	Upregulated	Plasma exosomes obtained from 40 patients with mesial TLE and 40 healthy controls	Diagnostic biomarker (mesial TLE patients vs controls)	–	–	0.8444	[64]
*miR-4668-5p*	Downregulated		Diagnostic biomarker (mesial TLE patients vs controls)	–	–	0.7894	
*miR-8071*	Downregulated		Diagnostic biomarker (mesial TLE patients vs controls)	83.33	96.67	0.9316	
*miR-197-5p*	Downregulated		Diagnostic biomarker (mesial TLE patients vs controls)	–	–	0.8017	
*miR-129–2-3p*	Upregulated	Brain tissues from 13 patients with refractory TLE and 13 healthy controls	Diagnostic biomarker (TLE patients vs controls)	–	–	0.929	[65]
*miR-129–2-3p*	Upregulated	Plasma samples from 25 patients with refractory TLE and 25 healthy controls	Diagnostic biomarker (TLE patients vs controls)	–	–	0.778	
*miR-106b-5p*	Upregulated	Serum samples from 147 epilepsy patients and 142 healthy individuals	Diagnostic biomarker (epilepsy patients vs controls)	80.3	81.2	0.882	[66]

### miRNAs polymorphisms in epilepsy

Association between single nucleotide polymorphisms within miR-146a and risk of epilepsy has been appraised in Brazilian, Chinese and Italian patients (Table 4). GC genotype of rs2910164 has been associated with higher risk of drug-resistant epilepsy among Brazilians. In addition, GC and CC genotypes of this SNP has been associated with low expression of miR-146a in epileptogenic tissues compared to GG genotype [67]. rs57095329 within this gene has also been correlated with risk of drug resistant epilepsy among Chinese patients [68]. In addition, A allele of rs57095329 has been associated with decreased frequency of seizures in drug resistant epilepsy patients [68]. However, rs2910164 has not been associated with risk of TLE among Italians [69].

**Table 4:** miRNAs polymorphisms in epilepsy

microRNA	Polymorphism	Samples	Population	Assay method	Results	Reference
*miR-146a*	SNP (rs57095329, rs2910164)	61 paraffin-embedded tissue specimens from patients with drug-resistant epilepsy and blood samples from 276 control subjects	Brazilian	TaqMan real-time PCR	rs2910164 GC genotype was associated with augmented risk of drug-resistant epilepsy. Also GC and CC genotypes of this SNP was associated with low expression of miR-146a in epileptogenic tissues compared to GG genotype	[67]
*miR-146a*	SNP (rs2910464, rs57095329)	Blood samples from 249 epilepsy patients and 249 healthy volunteers	Chinese	ABI PRISM SNapShot	rs57095329 was correlated with risk of drug resistant epilepsy. Also A allele of rs57095329 was associated with decreased frequency of seizures in drug resistant epilepsy patients	[68]
*pre-miR-146a*	SNP (rs2910164)	357 patients with TLE and 543 healthy individuals as controls	Italian	TaqMan allelic discrimination	There was no association between this variant and risk of TLE	[69]
*miR-146a*	SNP (rs57095329)	Blood samples from 267 childhood epilepsy patients and 267 age and gender matched normal individuals	Chinese	TaqMan allelic discrimination	rs57095329 polymorphism was associated with increased risk of drug-resistance development in epilepsy patients	[70]

## Discussion

miRNAs have been shown to affect several aspects of epliptogenesis. Modulation of apoptosis and survival of neurons and regulation of inflammatory responses are the most appreciated mechanisms of involvement of miRNAs in the pathogenesis of epilepsy. In addition to direct effects of miRNAs on molecular pathways in neurons, they can affect functions of reactive glial cells which potentially regulate inflammatory responses in the brain and remodeling of the extracellular matrix [26]. miR-132 and miR-146a are among the mostly assessed miRNAs in the animal models of epilepsy. These miRNAs have been found to affect several targets and pathways during epileptogenesis. For instance, miR-132 has interactions with TGF‐β and BDNF/TrkB signaling pathways in glial cells and neurons, respectively. miR-146a affects activity of Notch and NF-κB pathways in this context. SIRT1 and BDNF have been identified as molecular targets of several miRNAs in the context of epilepsy.

miRNAs have also diagnostic and prognostic functions in epilepsy. Some miRNAs such as miR-15a-5p, miR-328-3p, miR-129–2-3p and miR-106b-5p have been suggested as appropriate diagnostic markers in epilepsy, while miR-146 and miR-134 has been proposed as prognostic markers with mediocre performance. Moreover, miRNAs can modulate response of patients with refractory epilepsy to antiepileptic medications [6]. Therefore, miRNA-modulating therapeutic options might be used as alternative therapies for enhancing efficacy of antiepileptic drugs. Moreover, animal studies have shown that miRNA-targeting modalities might amend epilepsy-induced cognitive and behavioral impairments.

As the effects of miRNAs on glial cells and neurons might be exerted through different routes and even in different directions, miRNA-modulating therapies should be assessed in different cell types to validate their beneficial effects in each cell types.

## Conclusion

In brief, several miRNAs have been shown to be dysregulated in brain tissues and serum samples of patients with epilepsy and different animal models of this neurological condition. Abnormal levels of these miRNAs in the serum samples show their potential as biomarkers for prediction of epilepsy. However, the results of these studies should be verified in independent cohorts from different stages of epilepsy. Contribution of genetic variants within miRNA coding genes in risk of epilepsy or resistance to antiepileptic drugs is another research area which should be explored in future.

## Data Availability

Data sharing not applicable to this article as no datasets were generated or analysed during the current study.
